# Complete ACL injuries are associated with higher static tibial subluxation than partial ACL injuries or intact ACLs measured on MRI

**DOI:** 10.1002/jeo2.70526

**Published:** 2025-11-05

**Authors:** Johannes C. Harmes, Steffen T. Ubl, Evamaria Koch, Bertil Bouillon, Daniel Günther, Thomas R. Pfeiffer

**Affiliations:** ^1^ Department of Diagnostic and Interventional Radiology and Neuroradiology University Hospital Essen Germany; ^2^ Department of Orthopaedic Surgery, Trauma Surgery and Sports Medicine Cologne Merheim Medical Center Germany; ^3^ Department of Experimental Sports Traumatology Witten/Herdecke University Witten Germany

**Keywords:** anterior cruciate ligament, knee instability, magnetic resonance imaging, rotational instability, tibial subluxation

## Abstract

**Purpose:**

To investigate static tibial subluxation on magnetic resonance imaging (MRI) as a potential method to quantify rotatory knee instability and differentiate between complete anterior cruciate ligament (ACL) injuries, partial ACL injuries, and intact ACL.

**Methods:**

Consecutive patients who underwent knee arthroscopy between 2016 and 2021 were retrospectively reviewed and divided into two groups according to arthroscopically verified ACL status: partial (p‐ACL‐I) or complete ACL injury (c‐ACL‐I). Patients with p‐ACL‐I required ACL‐augmentation. The control group with an intraoperatively confirmed intact ACL (i‐ACL) and a meniscus injury was 1:1 matched to p‐ACL‐I considering age, sex and body mass index (BMI). Static tibial subluxation in the medial (MC) and lateral compartment (LC) was measured on preoperative MRI. Intraclass correlation coefficients (ICC) were calculated for inter‐ and intrarater reliability. Pairwise *t*‐test and multivariate logistic regression were used to identify differences with significance set at *p* < 0.05.

**Results:**

The final analysis included 136 patients, 30 patients with p‐ACL‐I (8 female, 28 ± 10 years, BMI 27 ± 5 kg/m^2^), 24 patients with an i‐ACL (6 female, 34 ± 11 years, BMI 28 ± 5 kg/m^2^) and 82 patients with c‐ACL‐I (26 female, 27 ± 11 years, BMI 26 ± 5 kg/m^2^). The static tibial subluxation was found to be greater in c‐ACL‐I (MC: 2.5 mm, LC: 6.8 mm) compared with the p‐ACL‐I (MC: −0.6 mm, LC: 1.7 mm) both in the MC and LC (*p* < 0.001). The static anterior tibial subluxation was greater in p‐ACL‐I (−1.1 mm) compared with i‐ACL (−4.1 mm) in the MC (*p* = 0.01). However, no significant difference between p‐ACL‐I (1.6 mm) and i‐ACL (0.9 mm) was found in the LC (*p* = 0.90). Intra‐ and interrater reliability analysis showed good to excellent agreement (ICC = 0.75–0.90).

**Conclusion:**

Residual fibres in p‐ACL‐I stabilized static anterior tibial translation. Preoperative static MRI subluxation measurement may help to differentiate between c‐ACL‐I and p‐ACL‐I who require surgical intervention, as an adjunct to the decisive dynamic examination of knee instability.

**Level of Evidence:**

Level III.

**Abbreviations**
ACL: anterior cruciate ligament
BMI: body mass index
c‐ACL‐I: complete ACL injury
i‐ACL: intact anterior cruciate ligament
ICC: intraclass correlation coefficient
Lat.: lateral
LC: lateral compartment
M: mean
Max.: maximum
MC: medial compartment
Med.: medial
Min.: minimum
mm: millimetres
MRI: magnetic resonance imaging
n.s.: not significant
p‐ACL‐I: partial ACL injury
PCL: posterior cruciate ligament
SD: standard deviation
WBCT: weight‐bearing computed tomography

## INTRODUCTION

One of the main symptoms of anterior cruciate ligament (ACL) injuries is the subjective feeling of instability, which can severely restrict the quality of life [28, 37, 44]. In particular, the degree of rotatory knee instability has a significant impact on diagnosis and treatment, but clinical quantification of the degree of rotatory knee instability is difficult [19, 22, 46, 48]. Even when using the pivot shift test, which is the most specific test for rotatory knee instability, there can be serious discrepancies between examiners due to different test design and the sensitivity of 48% [26, 43].

In order to quantify rotatory knee instability in a more standardised way, different methods including accelerometer, weight‐bearing computed tomography and image analysis technolgy have been developed [11, 14, 15, 24, 44, 51]. However, there is currently no widely implemented method for assessing rotatory knee instability in everyday clinical practice that provides a reliable assessment efficiently and with little additional time and financial cost. In partial ACL injuries (p‐ACL‐I), the decision between conservative and surgical treatment depends on knee instability [14]. But differentiating between a p‐ACL‐I requiring surgery and an intact ACL (i‐ACL) presents significant clinical and radiological challenges which is why the investigation of further diagnostic components for the assessment of p‐ACL‐I is of great interest [44, 47].

In recent years, the measurement of static tibial subluxation in relation to the femur using magnetic resonance imaging (MRI) has gained attention, as it is a cost‐effective, time‐saving and potentially conclusive method for the detection of rotatory knee instability. However, a comparison of static tibial subluxation in different arthroscopically confirmed ACL statuses is lacking.

The aim of the study was therefore (I) to assess the differences in static tibial subluxation between patients with p‐ACL‐I and those with an i‐ACL and (II) to assess the variations in static tibial subluxation between patients with complete ACL injuries (c‐ACL‐I) and those with p‐ACL‐I. It was hypothesized that there would be significant differences between c‐ACL‐I, p‐ACL‐I and i‐ACLs of the static tibial subluxation in the medial (MC) and lateral compartment (LC) measured on preoperative MRI.

## MATERIALS AND METHODS

**Figure 1:**
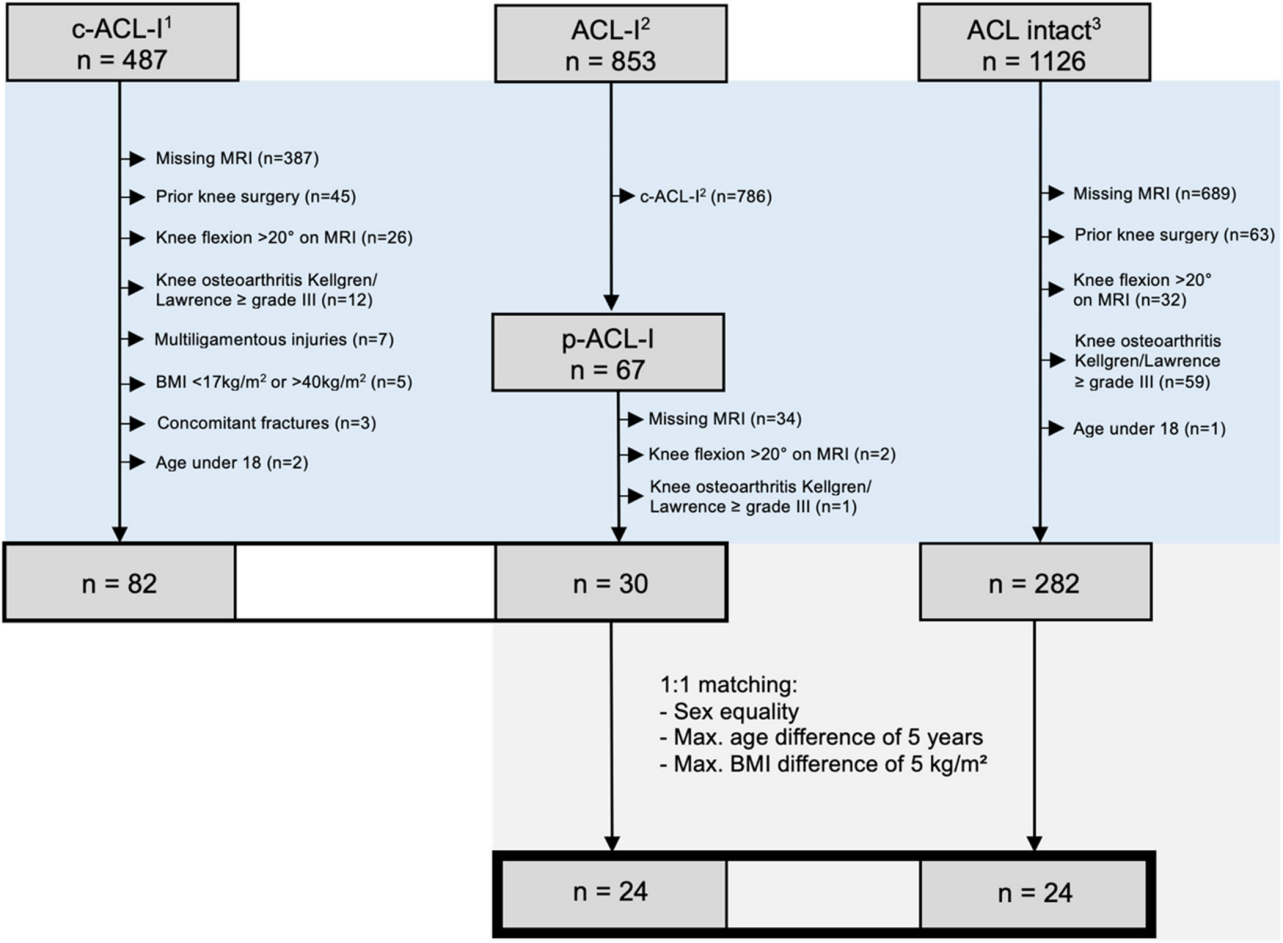
Patient collective. The respective cohort sizes and the number of patients (*n*) are illustrated. The inclusion criteria were arthroscopic knee surgery with evaluation of the ACL status and an available MRI scan. The exclusion criteria were age under 18, BMI under 17 kg/m^2^ or over 40 kg/m^2^, multiligamentous injuries, concomitant fractures, prior knee surgery, Kellgren/Lawrence ≥ grade III or a knee flexion >20° on MRI. The matching variables for the 1:1 matching were as follows: A maximum age difference of 5 years, a maximum BMI variance of 5 kg/m² and sex. The period of data collection was as follows: c‐ACL‐I^1^: 03/2018–06/2019; ACL‐I^2^, c‐ACL‐I^2^: 01/2019–02/2021; ACL intact^3^: 01/2016–02/2021. ACL, anterior cruciate ligament; ACL‐I, (all) ACL injuries; c‐ACL‐I, complete ACL injury; p‐ACL‐I, partial ACL injury; BMI, body mass index; MRI, magnetic resonance imaging; Max., maximum.

After obtaining approval from the Ethics Committee (102/2021) for our single‐center study, a retrospective analysis of consecutive patients who underwent surgical treatment of the knee from 2016 to 2021 was performed. Inclusion criteria were arthroscopic knee surgery with confirmation of the ACL status at our institution and the availability of preoperative magnetic resonance imaging (MRI). Patients were excluded if they were under 18 years of age, had a body mass index (BMI) under 17 kg/m^2^ or over 40 kg/m^2^ or multiligamentous injuries, concomitant fractures, prior knee surgery, knee osteoarthritis Kellgren/Lawrence ≥ grade III [20] or a knee flexion >20° on MRI (Figure 1). The most common reason for exclusion was the lack of the necessary image material. Our sample was divided into three groups according to the ACL status: p‐ACL‐I, c‐ACL‐I and i‐ACL. The arthroscopically verified ACL status was extracted from the surgical report. p‐ACL‐I was defined as a ruptured anterolateral or posterolateral bundle that was treated with ACL augmentation using an autograft, with the intact bundle being preserved. Patients of the i‐ACL group underwent isolated meniscal surgery without concomitant cartilage treatment and were matched to the patients with p‐ACL‐I considering age, sex and BMI. The criteria for this matching were an identical sex, a maximum age difference of 5 years and a maximum BMI deviation of 5 kg/m². In addition, p‐ACL‐I patients and patients with c‐ACL‐I were compared separately.

The clinic's internal programme ‘Centricity RA1000 Workstation’ (GE Healthcare) was used for the MRI measurements. For better visualization, the measurements used as examples in this paper were edited with the DICOM Medical Image Viewer HOROS programme (Version 3.3.5, Nimble Co LLC d/b/a; Purview).

**Figure 2:**
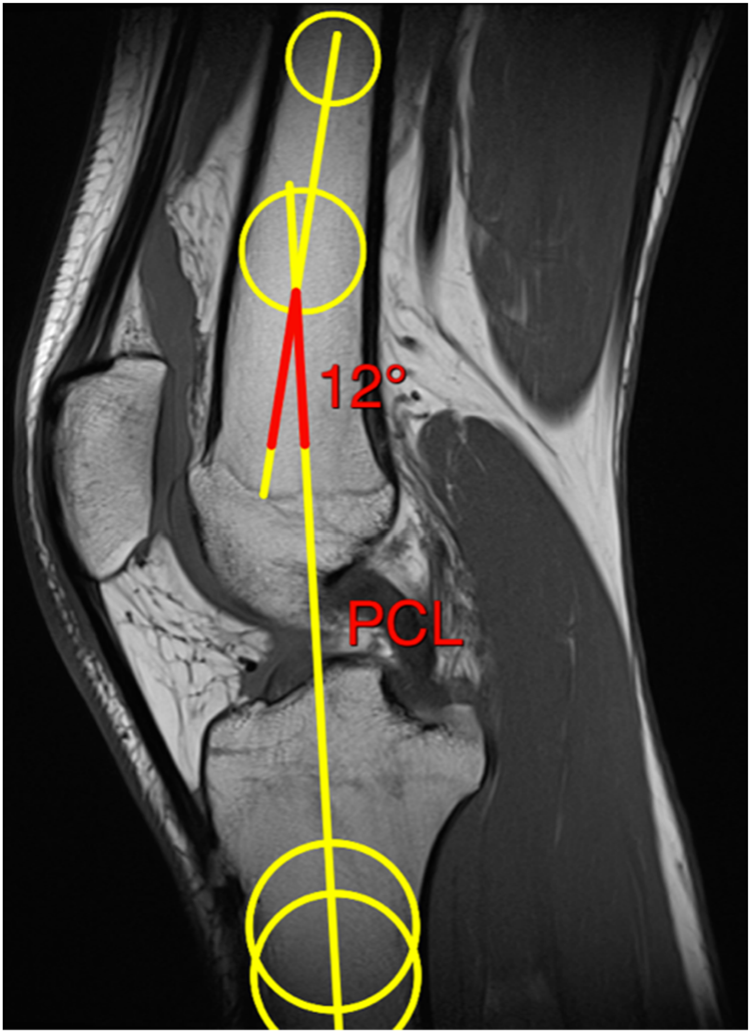
MRI flexion measurement. Sagittal T1‐weighted MRI images were used for the flexion measurement. The image slice was defined as the one on which the attachment of the PCL to the femur was visible. Two best‐fit circles were drawn in the subchondral portion of the femoral shaft, with the cortical bone of the femur serving as the limit of the circles. The first circle was placed as proximally as possible in the femoral shaft. The second circle was drawn in the distal part of the femoral shaft, whereby the center of this circle had to be at least 4 cm proximal to the femoral metaphysis. Next, two additional best‐fit circles were drawn in the proximal and distal parts of the tibia. The center points of these circles were connected by a line extending beyond them. The angle between the two resulting lines indicates the degree of flexion or extension of the knee joint. MRI, magnetic resonance imaging; PCL, posterior cruciate ligament.

Prior to the MRI subluxation measurement, the flexion angle of the knee joint was measured on the preoperative MRI (Figure 2). Although the patients were instructed to keep the leg as extended as possible during the MRI examination, full extension of the knee joint was often not possible due to concomitant injuries. Insufficient extension could have limited the comparability of the MRI subluxation measurements, as the angle of the knee joint influences the measurement of static tibial subluxation [27]. A flexion angle of more than 20° was used as an exclusion criterion, as the tibia rotates outwards during the transition to full extension, specifically during a movement of up to 20° flexion [18]. This movement causes the knee joint to lock due to the tension of the capsular‐ligamentous apparatus [18]. Sagittal T1‐weighted MRI images were acquired for the flexion measurement (Figure 2). The image slice was defined as the one on which the attachment of the posterior cruciate ligament (PCL) to the femur was visible (Figure 2). First, two best‐fit circles were drawn in the subchondral portion of the femoral shaft, with the cortical bone of the femur serving as the limit of the circles. The first circle was placed as proximally as possible in the femoral shaft. The second circle was drawn in the distal part of the femoral shaft, whereby the center of this circle had to be at least 4 cm proximal from the metaphysis of the femur. This was followed by the drawing of two further best‐fit circles in the proximal and distal parts of the tibia. The cortical bone again served as the limit of the circles. The center points of the ‘best‐fit circles’ for the femoral shaft and tibia were connected by a line and extended beyond these points. The angle between the two resulting lines indicates the degree of flexion or extension of the knee joint. Positive measured values indicated the flexion angle and negative values the extension angle.

**Figure 3:**
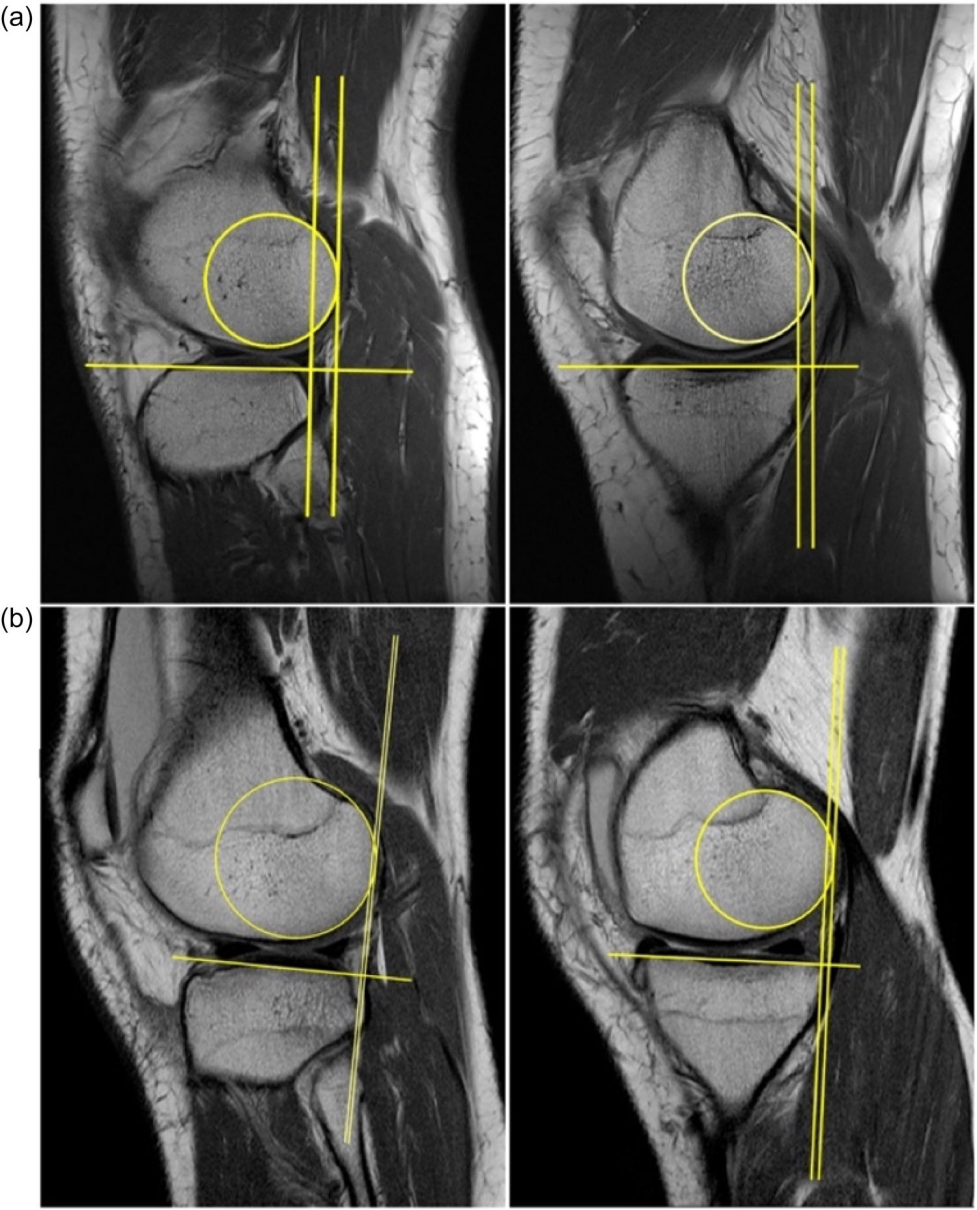
MRI subluxation measurements. Static anterior subluxation of the lateral (left) and medial (right) tibial plateau in patients with an i‐ACL and high (a) and patients with c‐ACL‐I and a low (b) rotatory knee laxity [27]. Preoperative sagittal T1‐weighted MRI scans of the patients' knees in the supine position were used to measure static tibial subluxation for all three cohorts in the MC and LC as previously described [27, 45]. The decisive factor for identifying the sectional image plane for the MC was the fermoral insertion of the medial gastrocnemius tendon and the most medial incision of the fibula in the tibiofibular joint for the LC. First, a best‐fit circle was placed over the subchondral portion of the posterior femoral condyle. Next, a straight line was drawn from the most anterior to the most posterior part of the tibial plateau and extended beyond the tibia. Another straight line was then drawn perpendicular to the previously line and tangentially approximated to the posterior edge of the best‐fit circle. Finally, a third straight line, also perpendicular to the tibial plateau, was drawn and approximated to the posterior side of the tibia. The resulting distance between the two parallel straight lines was defined as the degree of static tibial subluxation respectively translation. ACL, anterior cruciate ligament; i‐ACL, intact ACL; c‐ACL‐I, complete ACL injury; LC, lateral compartment; MC, medial compartment; MRI, magnetic resonance imaging.

Preoperative sagittal T1‐weighted MRI scans of the patients' knees in the supine position were used for the MRI evaluation and analyzed by measuring the static anterior tibial subluxation relative to the femur based on the method used by Tanaka et al., McDonald et al., Lian, Novaretti et al. and Zhang et al. [16, 27, 31, 45, 51]. All three cohorts were measured in the medial (MC) and lateral compartment (LC). The decisive factor for identifying the appropriate sectional image plane for the MC was the fermoral insertion of the tendon of the musculus gastrocnemius caput mediale [45]. For the measurement of the LC, the sectional image plane that showed the most medial incision of the fibula in the tibiofibular joint was selected [45]. The static tibial subluxation measurement was analogous in the MC and LC. The first step of the measurement method consisted of placing a best‐fit circle over the subchondral portion of the posterior femoral condyle [27]. Next, a straight line was drawn from the most anterior to the most posterior part of the tibial plateau and extended beyond the tibia [27] Another straight line was then drawn perpendicular to the previously line and tangentially approximated to the posterior edge of the best‐fit circle [27]. Finally, a third straight line, also perpendicular to the tibial plateau, was drawn and approximated to the posterior side of the tibia [27]. The resulting distance between the two parallel straight lines was defined as the degree of static tibial subluxation respectively translation [27] (Figure 3).

Both the extension measurement and the measurement of static anterior tibial subluxation were performed by two independent raters so that the interrater reliability could be determined. One was a radiology resident and the other was an orthopaedic surgery resident, who was the blinded observer. In addition, the radiology resident measured again after 14 days so that the intrarater reliability could be determined. The ‘two way mixed effect, absolute agreement, single rater model’ was used to interpret the ICC [13].

### Statistical analysis

Analyses were performed using IBM SPSS Statistics 29 (IBM). The propensity score matching was executed by R Statistical Software (R, version 4.3.2; R Foundation, https://www.R-project.org/). Descriptive statistics including means, standard deviations, minimums, and maximums were determined for all continuous variables.

Patients with p‐ACL‐I were matched to the i‐ACL control group considering age, sex and BMI using a 1:1 propensity score matching. The criteria for this matching were an identical sex, a maximum age difference of 5 years and a maximum BMI deviation of 5 kg/m². Pairwise *t*‐tests were performed for the parameters age, sex, BMI and the results of the MRI flexion measurement to verify patient matching. For the comparison of the static tibial subluxation measurements, pairwise *t*‐test and the effect sizes (Cohen's *d*) were calculated.

To compare the groups of p‐ACL‐I and c‐ACL‐I, variables were tested for normal distribution using the Shapiro–Wilk test. The Levene‐test, paired *t*‐tests with Bonferroni correction and boxplots were used to identify differences between the groups. Independent effect sizes (Cohen's *d*) were calculated for the comparison of the static tibial subluxation measurements. Multivariate logistic regression was used to assess the respective effect of age, sex, BMI and ACL status on the static tibial subluxation measured in MRI. The statistical significance was set at *p* < 0.05 for all calculations.

Two a priori sample size calculations were performed using G*Power 3.1.9.6 (HHU) for the respective comparisons. The power was set at 0.95 with an α‐error probability of 0.05. Due to the lack of literature comparing p‐ACL‐I and i‐ACL using MRI measurements, a mean effect size of 0.6 (Cohen's *d*) was assumed for two‐sided testing. Because p‐ACL‐I patients exhibited significantly greater rotatory instability than i‐ACL patients, indicating the need for surgery, a moderate to high effect size was assumed. The sample size calculation estimated that 39 patients (rounded up to 20 per group) would have to be included in the present study. For the second comparison, a required sample size of 29 partially ACL injured patients and 75 completely ACL‐injured patients was calculated with an expected effect size of Cohen's *d* = 0.8 and a distribution of the groups with a factor of 2.6, based on previous studies [8, 30, 51].

## RESULTS

A total of 136 patients, 30 patients with p‐ACL‐I (8 female, 28 ± 10 years, BMI 27 ± 5 kg/m^2^), 24 patients with an i‐ACL and a meniscus injury (6 female, 34 ± 11 years, BMI 28 ± 5 kg/m^2^) and 82 patients with c‐ACL‐I (26 female, 27 ± 11 years, BMI 26 ± 5 kg/m^2^) were included in the final analyses. No significant differences were found regarding age, sex, BMI and knee flexion within the groups (*p* > 0.01).

**Figure 4:**
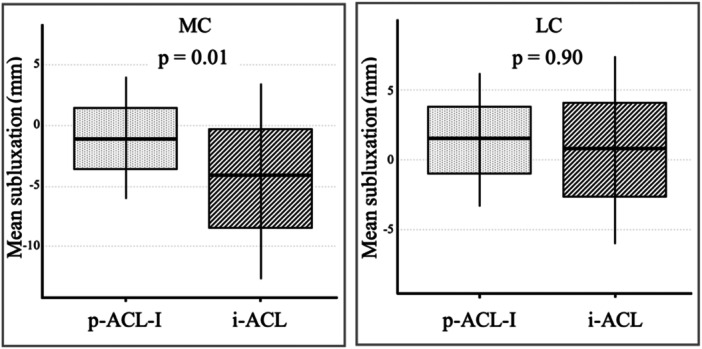
Partial ACL injury versus intact ACL. The mean values of the static anterior tibial subluxation relative to the femur in preoperative MRI measurements in patients with a partial ACL injury (grey, left) and the ACL intact control group (black, right) are shown in millimetres (mm) in the medial compartment (MC) and the lateral compartment (LC) with the corresponding standard deviation. ACL, anterior cruciate ligament; i‐ACL, intact ACL; MRI, magnetic resonance imaging; p‐ACL‐I, partial ACL injury.

The matching of the p‐ACL‐I and the i‐ACL resulted in 24 pairs (Table 1). The mean static anterior tibial subluxation in the MC was significantly greater (*p* = 0.01, 95% CI [−5.1; −0.99], *d* = −0.63) in p‐ACL‐I (−1.1 ± 2.7 mm) compared with i‐ACL (−4.1 ± 3.9 mm). However, no significant difference (*p* = 0.90, 95% CI [−2.4; 1.1], *d* = −0.16) was found in the LC (p‐ACL‐I: 1.6 ± 2.7 mm; i‐ACL: 0.9 ± 3.6 mm) (Figure 4).

**Table 1:** Partial ACL injury versus intact ACL.

	p‐ACL‐I (*n* = 24)				i‐ACL (*n* = 24)				*p*‐Value
	*M*	SD	Min.	Max.	*M*	SD	Min.	Max.	
Age (years)	28	10	14	54	34	11	21	40	*p* = 0.26
BMI (kg/m²)	27	5	20	37	28	5	21	40	*p* = 1.00
Sex	Female: 6 male: 18				Female: 6 male: 18				*p* = 1.00
Extension/flexion angle (°)	8	7	−10	19	7	7	−6	19	*p* = 1.00
MC (mm)	−1.1	2.7	−6.1	4	−4.1	3.9	−12.7	3.4	*p* = 0.01
LC (mm)	1.6	2.7	−3.3	6.3	0.9	3.6	−6	7.5	*p* = 0.90

*Note*: The mean values (*M*) of the static anterior tibial subluxation relative to the femur in preoperative MRI measurements in patients with a partial ACL injury and the intact ACL control group are shown in millimetres in the medial (Med.) and the lateral (Lat.) compartment with the corresponding standard deviation (SD), minimum (Min.) and maximum (max.). A negative angle indicates extension and a positive angle indicates flexion.

Abbreviations: ACL, anterior cruciate ligament; i‐ACL, intact ACL; LC, lateral compartment; MC, medial compartment; MRI, magnetic resonance imaging; *n*, number; p‐ACL‐I, partial ACL injury.

The ICC was 0.95 (95% CI [0.88–0.98]) for the MC of the p‐ACL‐I and 0.98 (95% CI [0.96–0.99]) for the LC. For the c‐ACL‐I, the ICC was 0.88 (95% CI [0.77–0.93]) in the MC and 0.92 (95% CI [0.87–0.94]) for the LC. In i‐ACL patients, the ICC was 0.91 (95% CI [0.83–0.94]) in the MC and 0.94 (95% CI [0.89–0.98]) for the LC. The standard error of the mean was 0.5 mm for patients with p‐ACL‐I in both the MC and LC. For c‐ACL‐I, it was 0.3 mm in the MC and 0.4 mm in the LC. For i‐ACL, it was 0.8 mm in the MC and 0.7 mm in the LC.

**Figure 5:**
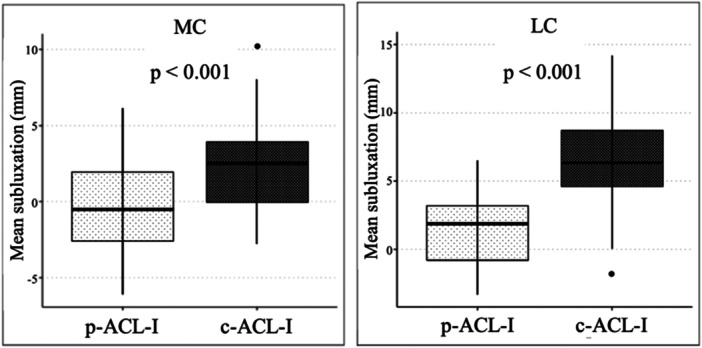
Partial ACL injury versus complete ACL injury. The mean values of the static anterior tibial subluxation relative to the femur in preoperative MRI measurements in patients with a partial ACL injury (grey, left) and a complete ACL injury (black, right) are shown in millimetres (mm) in the medial (MC) and the lateral compartment (LC) with the corresponding standard deviation. ACL, anterior cruciate ligament; c‐ACL‐I, complete ACL injury; p‐ACL‐I, partial ACL injury.

The static tibial subluxation in the MC was found to be significantly greater (*p* < 0.001, 95% CI [2.0; 4.2], *d* = 1.17) in c‐ACL‐I (2.5 ± 2.5 mm) compared with the p‐ACL‐I (−0.6 ± 2.9 mm). Moreover, the static tibial subluxation in the LC was found to be greater (*t*(111) = 7.02; *p* < 0.001, 95% CI [3.7; 6.5], *d* = 1.5) in c‐ACL‐I (6.8 ± 3.6 mm) compared with p‐ACL‐I (1.7 ± 2.8 mm) (Figure 5).

These results were confirmed by multivariate regression analysis under consideration of age, sex and BMI. A significant influence of the ACL injury pattern, adjusted for the influences of the confounders, was demonstrated for both the MC (*t* = 5.2, *p* < 0.001) and the LC (*t *= 7, *p* < 0.001). The influence of the ACL injury pattern was the greatest among the variables tested (medial: beta = 0.45; lateral: beta = 0.54). Intra‐ and Interrater reliability analysis showed good to excellent agreement (ICC = 0.75–0.90) for the MRI‐based measurement of the static tibial subluxation.

## DISCUSSION

The mean static anterior tibial subluxation relative to the femur was significantly different between patients with p‐ACL‐I and c‐ACL‐I in both the MC and LC. There was greater static tibial subluxation in patients with c‐ACL‐I. It can be concluded for surgical therapy that the residual fibres of a p‐ACL‐I have a stabilizing function. This finding is consistent with the results of previous studies [15] and can be used as an argument in favour of ACL augmentation, although it is technically complex, and against alternative ACL reconstruction in patients for whom surgery is indicated due to clinically assessed knee instability [50]. In addition to the mechanical reasons shown in this study, ACL augmentation is preferred over ACL reconstruction for vascular and proprioceptive reasons [1, 6, 8, 12, 35, 36].

MRI measurement of static anterior subluxation of the tibia relative to the femur might also be a potential adjunct to the essential dynamic evaluation of knee stability. In the past authors have criticized the lack of a gold standard for the assessment of rotational knee stability [24, 29, 34]. Various approaches have been developed in the past to measure knee instability in a more standardized way [8, 11, 15, 24, 27, 40, 51]. Examples include the establishment of a standardized procedure for clinical tests, for example the pivot shift test [29, 34], a dynamic tibial subluxation measurement using an arthrometer [9, 45], the use of stress radiographs [4, 45] or weight‐bearing computed tomography (WBCT) [24]. These methods have advantages and disadvantages with regard to the predictive value of the tibial subluxation and the clinical applicability [4, 9, 10, 27, 45]. For example, although the arthrometer offers the possibility of measuring dynamic tibial subluxation largely independent of the examiner's experience, and has thus been able to demonstrate repeatable data in clinical trials, the feasibility, affordability, additional time and effort, and limited patient comfort compared to the static MRI measurement argue against the use of an arthrometer [32, 38, 49].

Another approach to the quantification of tibial subluxation is the use of a pivot‐shift app, partly with implemented machine learning algorithms, in which the influence of the individual examination performance of the traditional test is to be reduced, which should also lead to a reproducible measurement of dynamic tibial subluxation with the advantage of easier portability compared to the arthrometer and the disadvantages, compared to the MRI measurement of our study, in terms of the remaining individual examination performance, the increased technological dependence on external hardware, the lack of long‐term results, the requirement for regular calibration and the associated time expenditure [5, 38]. Due to the technical progress in machine learning and improved hardware equipment, the app‐based solution could become more relevant in the future. A prior study successfully used an app to quantify the medial rotatory instability of the knee [48]. So far, however, none of these methods became established universally in everyday clinical practice. It can, therefore, remain difficult to quantify the knee instability [34, 46]. The quick‐to‐use static measurement on existing MRI images investigated in this study represents an effective addition to the clinical examination.

The static anterior tibial subluxation in the MC measured in preoperative MRI was significantly greater in patients with p‐ACL‐I compared to patients with i‐ACLs. Considering that the p‐ACL‐I patients were operated due to dynamic instability in the clinical examination, this suggests that MRI measurement of the static tibial subluxation in the MC might perspectively contribute to the differentiation of p‐ACL‐I with significant instability and i‐ACL, especially once cut‐off values have been defined. Dynamic examination will remain the most important component of ACL diagnostics, but it is conceivable that in clinically unclear cases, examination of static knee stability could play a role in addition to the decisive dynamic knee stability.

Previously published studies using the same MRI measurement method to quantify static tibial subluxation in patients with an i‐ACL showed comparable results, taking into account differences in study design, in particular the different knee position in these previous studies and the clinical or radiological verification of ACL status compared with the intraoperative verification of ACL status in our study [7, 45, 51]. This as well as the almost perfect inter‐ and intrarater agreement (ICC 0.88–0.98) in our study indicate a fundamentally consistent method of measuring static tibial subluxation [23]. To the best of our knowledge, no studies have been conducted in which static tibial subluxation was investigated using the measurement method used in this study in patients with p‐ACL‐I in terms of arthroscopically confirmed residual fibres as previously described.

It is noticeable that the static tibial subluxation in the MC but not in the LC differed significantly between patients with a p‐ACL‐I and i‐ACL patients. One reason for this could be the higher muscular reinforcement of the lateral compartment, for example, by the iliotibial tract. Assuming that the status of the meniscus had an influence on the static tibial subluxation, a greater medial subluxation could be present on average in p‐ACL‐I, as the medial meniscus is significantly more frequently affected in traumatic injuries (75%) than the lateral meniscus (25%) [21].

Taking into account the results of the previous studies, the knee joint position during the MRI scan had a significant influence on the measurement of static tibial subluxation. To avoid falsification, the flexion of the knee joint was measured in our study and patients with a flexion of more than 20° were excluded. In preliminary studies, an attempt was made to achieve the best possible extension of the knee joints by positioning the knee joints using devices like foam blocks and positioning them in a knee coil [27, 31, 45]. A control of the success of this procedure by measuring flexion was not described. In our study, however, the patients were only asked to keep the leg in the best possible extension during the MRI examination, which is considered standard for knee MRIs [17]. In combination with the subsequent flexion measurement, the advantage of is that it is easier to apply in everyday clinical practice and still reliably excludes patients with an excessively flexed knee joint during the MRI examination.

Furthermore, correctly identifying the ACL status was essential for comparing our study with previous publications. In our study, the ACL injury pattern was determined exclusively intraoperatively, which is considered the gold standard. However, in previous studies investigating static tibial subluxation on MRI, ACL status was verified radiologically or clinically [31, 45, 52]. This could have led to potential misidentification of the ACL injury patterns, resulting in limited comparability of static tibial subluxation values in our study.

Finally, MRI measurement of the static anterior subluxation might be used as an adjunct to the decisive dynamic clinical examination. Due to the time required to measure flexion and static tibial subluxation, a fully automated machine learning based application would be valuable. Further studies with the implementation of corresponding algorithms and definition of cut‐off values for the differentiation between p‐ACL‐I, c‐ACL‐I and i‐ACL would be desirable.

### Limitation

One limitation to this study was that a number of patients were excluded because no MRI was available and because of the intention to achieve the best possible matched‐pairs in terms of age, sex and BMI. This resulted in patient exclusion due to age in particular, as the i‐ACL cohort consists of patients with a meniscus injury, who are older on average [25].

The different positions of the knee joint during the MRI scan were another limitation, as the angle of knee flexion during the MRI scan can affect the static tibial subluxation measurement [27]. Excluding patients with more than 20° of flexion reduced the influence of knee position on static tibial subluxation measurements, but may not have eliminated it completely. In addition, different MRI scanner and slice thicknesses were used, which can also influence the assessment [39]. On the other hand, this may also be an advantage in terms of the transferability of the results to everyday clinical measurements.

The arthroscopically verified ACL status was extracted from the surgical reports. This was a limitation because the assessment was subjective and may vary from surgeon to surgeon.

Potential weaknesses of the measurement method included the difficulty of drawing a straight line that is exactly tangent to the convex surface of the lateral tibial plateau, the variability in the size and shape of the femoral condyles, and the sometimes difficult identification of the correct slice level [45, 51]. However, the good to excellent intra‐ and interrater reliability of this study did not suggest a systematic measurement error [23]. Moreover, there is no consensus on whether meniscal injury and cartilage status affect the static tibial subluxation, and therefore the cartilage and meniscal status were not assessed in this study [2, 3, 27, 31, 41, 42, 45].

Finally, the i‐ACL group consisted of patients with an isolated meniscal injury, which may have influenced the static tibial subluxation. There is no consensus on whether meniscus injuries affect static knee stability [2, 27, 31, 33, 42]. One study found that static anterior tibial subluxation was significantly greater in patients with medial meniscal tears than in patients with intact menisci [42]. Conversely, some studies found no influence of meniscal injuries on knee stability in either the MC or the LC [3, 45]. Finally, it remained uncertain whether and to what extent the meniscal injury in the i‐ACL group influenced the static anterior subluxation. This possible influence was tolerated in favour of intraoperative confirmation of the intact ACL status.

## CONCLUSION

Residual fibres in p‐ACL‐I stabilized the static anterior tibial translation. Preoperative static MRI subluxation measurement may help to differentiate between p‐ACL‐I and c‐ACL‐I, as an adjunct to the decisive dynamic examination of knee instability. The measurement of the MC might also help to distinguish between i‐ACL and p‐ACL‐I with significant static instability. However, cut‐off values should be determined in further studies.

## AUTHOR CONTRIBUTIONS

All authors contributed to the study conception and design. Material preparation and data collection were done by Johannes C. Harmes, Steffen T. Ubl and Evamaria Koch. Measurements were performed by Johannes C. Harmes, Steffen T. Ubl and Evamaria Koch. The first draft of the manuscript was written by Johannes C. Harmes. Thomas R. Pfeiffer and Steffen T. Ubl made meaningful corrections to the structure of the article and guided the statistical methods and data processing. All authors commented on previous versions of the manuscript. All authors have read and approved the manuscript.

## CONFLICT OF INTEREST STATEMENT

The authors declare no conflict of interest.

## ETHICS STATEMENT

Positive ethics vote by the Ethics Committee of Witten/Herdecke University on 03.11.2021 (approval number: 102/2021).

## Data Availability

The data that support the findings of this study are available from the corresponding author upon reasonable request.
